# Hemostatic efficacy of two topical adjunctive hemostats in a porcine spleen biopsy punch model of moderate bleeding

**DOI:** 10.1007/s10856-021-06586-8

**Published:** 2021-09-30

**Authors:** Melinda H. MacDonald, Gary Zhang, Laura Tasse, Daidong Wang, Hector De Leon, Richard Kocharian

**Affiliations:** 1 Ethicon, Inc., Johnson & Johnson, US Highway 22 West, Somerville, NJ 08876-0151 USA; 2 NAMSA, 6750 Wales Rd, Northwood, OH 43619 USA; 3 Cardiovascular and Specialty Solutions (CSS), Johnson & Johnson, 29A Technology Dr, Irvine, CA 92618 USA; 4 Scientific Consultant, 184 Bonita Hills Rd, Athens, GA 30605 USA

**Keywords:** Hemostasis, Collagen, Thrombin, Chondroitin sulfate, Topical flowable hemostats

## Abstract

Topical hemostatic agents have become essential tools to aid in preventing excessive bleeding in surgical or emergency settings and to mitigate the associated risks of serious complications. In the present study, we compared the hemostatic efficacy of SURGIFLO^®^ Hemostatic Matrix Kit with Thrombin (Surgiflo—flowable gelatin matrix plus human thrombin) to HEMOBLAST™ Bellows Hemostatic Agent (Hemoblast—a combination product consisting of collagen, chondroitin sulfate, and human thrombin). Surgiflo and Hemoblast were randomly tested in experimentally induced bleeding lesions on the spleens of four pigs. Primary endpoints included hemostatic efficacy measured by absolute time to hemostasis (TTH) within 5 min. Secondary endpoints included the number of product applications and the percent of product needed from each device to achieve hemostasis. Surgiflo demonstrated significantly higher hemostatic efficacy and lower TTH (*p* < 0.01) than Hemoblast. Surgiflo-treated lesion sites achieved hemostasis in 77.4% of cases following a single product application vs. 3.3% of Hemoblast-treated sites. On average, Surgiflo-treated sites required 63% less product applications than Hemoblast-treated sites (1.26 ± 0.0.51 vs. 3.37 ± 1.16). Surgiflo provided more effective and faster hemostasis than Hemoblast. Since both products contain thrombin to activate endogenous fibrinogen and accelerate clot formation, the superior hemostatic efficacy of Surgiflo in the porcine spleen punch biopsy model seems to be due to Surgiflo’s property as a malleable barrier able to adjust to defect topography and to provide an environment for platelets to adhere and aggregate. Surgiflo combines a flowable gelatin matrix and a delivery system well-suited for precise application to bleeding sites where other methods of hemostasis may be impractical or ineffective.

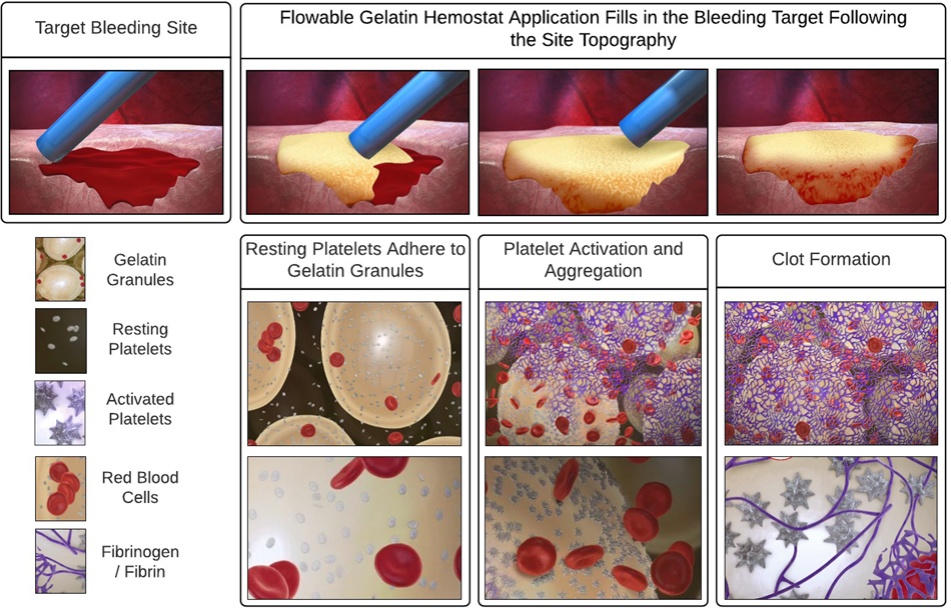

## Introduction

Hemostasis is a multi-layered cellular and molecular response to stop hemorrhage at the site of tissue injury. Primary hemostasis is the result of platelet activation following direct damage to vascular tissue. Platelets aggregate and adhere to the exposed subendothelial tissue in response to locally released factors (e.g., thromboxane A_2_, serotonin, and adenosine diphosphate), which also induce local vasoconstriction to prevent further bleeding [1]. Collagens and other subendothelial and perivascular extracellular matrix (ECM) proteins including vitronectin, laminin, and fibronectin facilitate platelet adhesion and activation [2]. Activation of the coagulation cascade, referred to as secondary hemostasis, occurs via the intrinsic (contact activation) and extrinsic (tissue factor-mediated) enzymatic pathways. The clotting cascade consists of a series of zymogens proteolytically converted to active forms that converge to induce the formation of a fibrin clot, a three-dimensional network of polymerized fibrin that provides additional strength to the original hemostatic plug composed mainly of aggregated platelets [3].

The ability to accelerate hemostasis at surgical sites is a factor of paramount importance to avoid blood loss, reduce perioperative morbidity and operative times and improve surgical outcomes [4]. Over 17 million therapeutic, invasive surgeries were reported in the United States (US) during 2014 [5]. These numbers have steadily increased since 1992 as reported by the National Ambulatory Medical Care Survey [6, 7]. A retrospective analysis of over 1.5 million surgeries in the US alone during 2006–2007 reported blood loss-related complications and blood product transfusions in nearly 30% and 21% of patients, respectively, which resulted in longer lengths of stay and higher total hospital costs [8]. A recent prospective analysis of 1459 patients indicated that the cost of cardiac surgery was 1.76 (CI, 1.64–1.90) times higher for patients that had a bleeding event since they are more likely to need additional procedures and have other adverse outcomes than patients that did not experience a bleeding event [9].

Novel topical hemostatic matrices containing a variety of biological products have been developed for use in open surgeries and laparoscopic procedures over the past two decades [10–17]. Adjunctive hemostats used by surgical teams may contain various agents such as gelatins, fibrin sealants, oxidized regenerated cellulose (ORC), or synthetic hydrogels and adhesives [18, 19]. Products based on animal ECM gelatins (e.g., porcine, bovine) and plant-derived ORC are at the clinical forefront of bleeding management because they can be used in a variety of surgical procedures with minimal risk of adverse effects [20, 21]. Delivery devices containing topical hemostats have been approved by the FDA in the US for surgical procedures when controlling bleeding by conventional procedures including pressure, suturing, and ligation is considered either ineffective or impractical [19, 21].

Flowable gelatin-based products are highly efficacious topical hemostats consisting of matrices that form gels in the presence of 0.9% NaCl solution [19, 22]. To accelerate coagulation in situ, thrombin, a terminal serine protease of the coagulation cascade, may be added. The aim of the present study was to generate in vivo evidence of the efficacy of two topical adjunctive hemostats, SURGIFLO^®^ Hemostatic Matrix Kit with Thrombin (Surgiflo), and HEMOBLAST™ Bellows Hemostatic Agent (Hemoblast). To that end, the study objective was to compare the in vivo hemostatic efficacy of Surgiflo and Hemoblast using a well-accepted animal bleeding model. Surgiflo is a single-use, gelatin-based flowable hemostat that forms a matrix upon reconstitution and can be used up to 8 h after mixing with human thrombin [23]. Hemoblast contains a powder combination product consisting of collagen, chondroitin sulfate, and human thrombin [24]. We tested the specific hypothesis that Surgiflo is as efficacious a topical adjunctive hemostat as Hemoblast in an in vivo porcine spleen biopsy punch model of bleeding. The animal model was selected based on the similarities between experimental bleeding of organs of large mammalian species and surgically induced bleeding in humans [25]. The interventional nature of in vivo large animal studies offers the advantage of establishing causal relationships and a tighter control of the variables considered in human clinical studies, including surgery type, age, sex, weight, and comorbidities. Similar, tightly controlled human studies designed to assess the efficacy of topical adjunctive hemostats, are expensive and impractical endeavors that may result in non-generalizable knowledge to larger populations—low external validity.

## Materials and methods

### Test materials

The two adjunctive hemostasts tested in the study included Surgiflo and Hemoblast. Surgiflo consists of a porcine gelatin paste supplied in a pre-filled syringe that can be mixed with human thrombin provided as lyophilized powder ready for reconstitution. The original Surgiflo (SURGIFLO Haemostatic Matrix plus FlexTip) launched in the US in 2005 did not include thrombin; however, as indicated in the instructions for use (IFU), the product could and frequently was used with thrombin added by the end users [26]. The Surgiflo matrix used in the present study consists of a reformulated gelatin-based flowable paste with higher solid content and a higher degree of gelatin cross-linking, enhanced viscosity, consistency, and usability. Available in the US since 2012 [23], the Surgiflo matrix was provided in a kit format with lyophilized human thrombin (SURGIFLO^®^ Hemostatic Matrix Kit), which was reconstituted and mixed with the matrix according to the IFU. Surgiflo is manufactured by Ferrosan Medical Devices (Soborg, Denmark) and distributed by Ethicon, Inc. (Somerville, New Jersey, USA) in the USA. Thrombin (Evithrom, human plasma-derived thrombin) is produced by Omrix Biopharmaceuticals (Kiryat Ono, Israel) and distributed by Ethicon, both Johnson & Johnson (New Brunswick, New Jersey, USA) companies. Hemoblast-B is a hemostatic product consisting of porcine-derived collagen, bovine chondroitin sulfate, and human-derived thrombin pre-loaded as powder in a bellows for administration [27]. Hemoblast is manufactured by Biom’up SA (Lyon, France).

Both Surgiflo and Hemoblast are hemostatic products indicated for adjunctive management of diffuse or localized bleeding areas and are delivered after blotting excess blood from the target sites. Both adjunctive hemostats were provided in sterile packages and used according to the manufacturer’s instructions. Surgiflo was prepared by reconstituting 2000 international units (IU) of human thrombin in 2 ml of sterile water followed by mixing with the gelatin matrix that resulted in a final volume of 8 ml. Hemoblast is supplied as a bellows pre-loaded with hemostatic powder (1.65 g). A 10 cm nozzle extension is included to assist with powder application. Each bellows device contains a maximum of 1500 IU of human thrombin. Test devices were weighed before and after application of the test material to each defect site in order to determine the total mass of article used in single or multiple applications required to treat each site.

### Product application

The Hemoblast product used in this study is approved for use and marketed in the European Economic Area (EEA, Conformité Européene [CE Marking], 2016) [24]. The same product has also been approved in the United States (US) (Food and Drug Administration [FDA], 2017) [28]. However, Hemoblast is marketed in the US using labeling information somewhat different from that of the product marketed in the EEA [27]. Specifically, the IFU provided with Hemoblast in the US recommends that a gauze or laparotomy pad soaked with saline be used to hold the hemostatic material at the target bleeding site for ~3 min prior to inspecting the area. In contrast, the IFU of the CE Marking product used in the present study instructs users to hold the hemostatic powder in contact with the bleeding area for at least 2 min. Surgiflo’s IFU instructs users to apply a saline moistened gauze over the matrix for 1–2 min before inspecting the wound site [23].

**Fig. 1:**
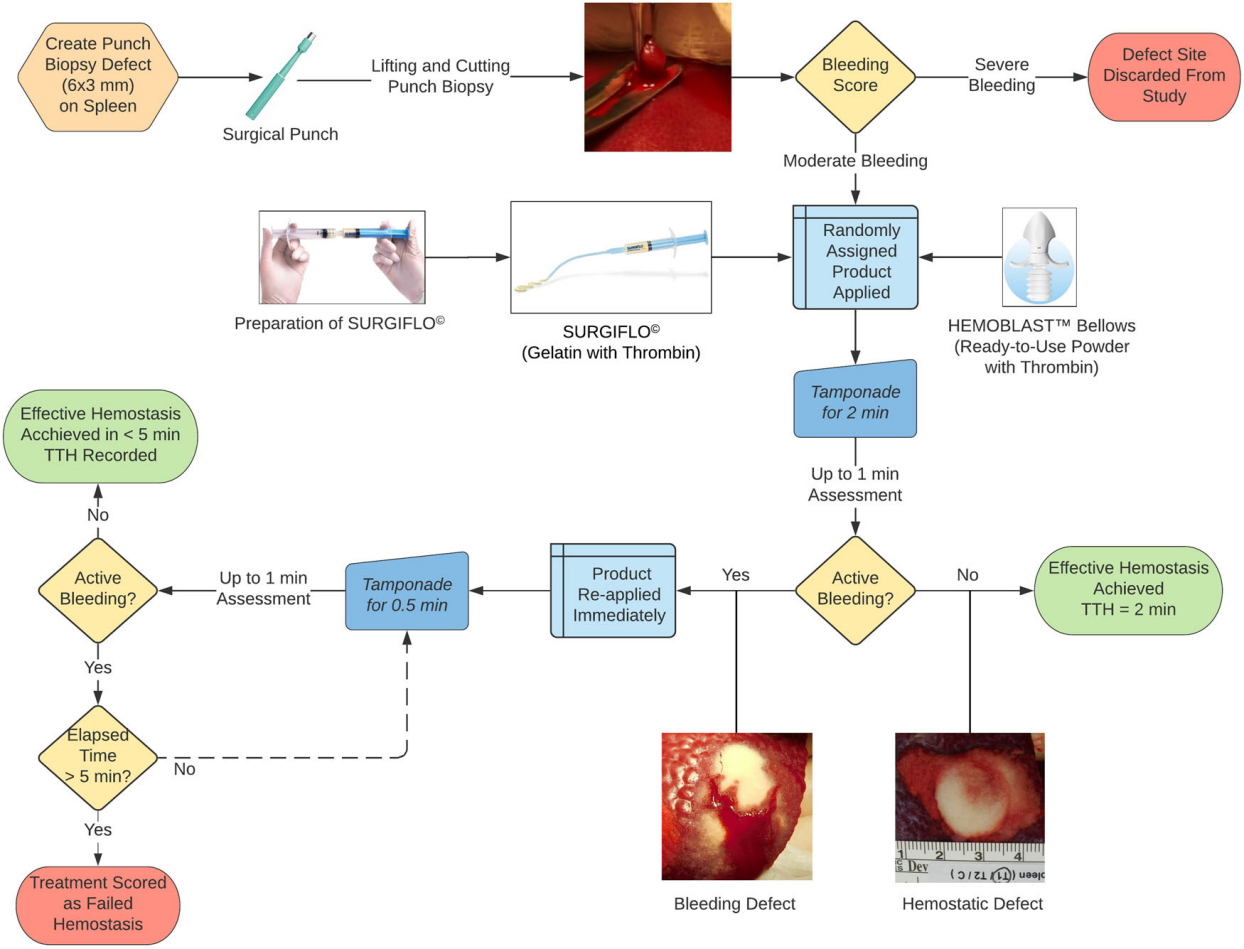
Schematic illustration of the porcine spleen hemostasis model. After a punch biopsy defect is created and bleeding scored, randomly assigned test material (Surgiflo or Hemoblast) and tamponade are applied for 2 min and evaluated for up to 1 min. If hemostasis is achieved following this initial 2-min period of tamponade and maintained for the full 1-min assessment period, time to hemostasis (TTH) is recorded as 2 min. If active bleeding is presentat any time during the up to 1-min assessment period, additional test material and tamponade are immediately applied for 0.5 min followed by another up-to-1-min assessment time. Subsequent 0.5 min product/tamponade and up to 1-min assessment periods are repeated until hemostasis is achieved or the 5-min endpoint period is reached without achieving hemostasis (failed hemostasis)

Both Surgiflo and Hemoblast were applied to the splenic lesions following the manufacturer’s IFU supplied with the products tested regarding initial tamponade. Briefly, Hemoblast powder or Surgiflo matrix was applied and compressed against the target bleeding site for 2 min using a moist surgical gauze and then the site was observed for evidence of continued bleeding (Fig. 1). As instructed in the IFU for each product, enough material to fully cover the entire bleeding area was used. To reflect a realistic clinical use scenario, our study design consisted of assessing hemostatic efficacy within 5 min after the initial application of the materials tested. Therefore, if hemostasis was not achieved after the initial 2-min period of tamponade, full 2-min compression periods were not repeated for either product. Instead, additional test material was applied followed by 30-s compression and visual assessment periods (see “Treated sites” section for further details).

### Animals

Female, nulliparous Yorkshire swine weighing 78–81 kg (5 months old) were used for the hemostasis test. Animals were ear-tagged and acclimated at the testing site for at least 3 days before any procedure was conducted. Animals were individually housed, fed once a day with standard pig chow and had access to water ad libitum. Housing and husbandry conformed to the standards of the “Guide for the Care and Use of Laboratory Animals” [29]. The study was conducted by NAMSA (Northwood, OH, USA) in their AAALAC International accredited facility. Animal protocols were reviewed and approved by NAMSA’s Institutional Animal Care and Use Committee (IACUC) and followed the set of recommendations included in the ARRIVE (Animal Research: Reporting of In Vivo Experiments) guidelines (ARRIVE Essential 10) [30]. The animal protocol considered the following inclusion/exclusion criteria. Only healthy animals were included in the study. Animals that were found to have splenomegaly before surgery or intraoperatively were excluded from the study. Only mild and moderate spleen bleeding lesions (see “Porcine hemostasis model” section) were included in the study. If a spleen defect site exhibited severe, uncontrolled bleeding, the animal would be humanly euthanized immediately, excluded from the study, and replaced. If any other intraoperative, unmanageable complication occurred, the animal would be euthanized while under anesthesia. No animal was euthanized for these reasons.

#### Porcine hemostasis model

An in vivo porcine spleen biopsy punch model was utilized to compare the hemostatic performance of the test materials [31]. Animals were anesthetized with intramuscular injections of a combination of tiletamine (3 mg/kg), zolazepam (3 mg/kg) (Telazol^®^), and xylazine (2.2 mg/kg), followed by endotracheal intubation and maintenance of anesthesia by inhalation of isoflurane (1–4%) mixed with 100% oxygen (~1.5 L/min). In addition to general anesthesia, the experimental animals received analgesic medication prior to the surgical intervention (buprenorphine, 0.02 mg/kg, intramuscular). Mechanical ventilation (10–20 ml/kg, 10–15 respirations/minute) was used throughout the procedure. The marginal ear vein of each pig was catheterized for delivery of fluids and drugs as needed. Lactated Ringer’s solution was administered intravenously (IV) at a rate of 5–10 ml/kg/h throughout the surgical procedure. Blood pressure was measured in the carotid artery using a catheter connected to a transducer. Heart and respiratory rates (HR, RR), electrocardiography (ECG), CO2, body temperature, and mean arterial blood pressure (MAP) were monitored continuously. The fluid infusion rate was adjusted as needed to maintain physiologic blood pressure levels prior to defect creation. A dobutamine HCl solution (0.3 mg/mL) was administered IV as required to ensure that MAP was maintained within 60–90 mmHg.

The porcine hemostasis model used in this study has been described previously [31]. Briefly, the abdominal cavity was opened through a ventral midline incision. The spleen was located and progressively exposed throughout the procedure until all biopsy defect sites were created and materials tested. The exposed spleen was kept moist continuously using saline-soaked gauzes. Integra, Miltex (Integra Life Sciences, Princeton, NJ, USA) 6-mm surgical biopsy punches were used to create similarly sized bleeding defects. Twenty-two to 24 biopsy-punch bleeding sites per pig (*n* = 3) were created on the surface of the spleen beginning at its distal end. Three additional replacement sites were created in a fourth animal for a total of 30 Hemoblast-treated and 31 Surgiflo-treated-sites (Table 1). Each biopsy measured ~6 mm in diameter by 3 mm in depth. A hemostasis scoring scale consisting of three levels, mild (capillary, arteriole, or venule oozing), moderate (flowing venous and/or arterial bleeding), and severe (pulsatile arterial or high-volume venous bleeding) bleeding was used to score each lesion. If a lesion was scored as “severe” bleeding, the defect site was excluded from testing and bleeding controlled by alternate surgical techniques (e.g., tamponade, clamping). Defect sites were treated sequentially. After testing and data acquisition at each site was completed, the surgeon ensured that hemostasis was achieved prior to creating the next defect site.

**Table 1:** Experimental design to test the efficacy of Surgiflo and Hemoblast in a porcine spleen biopsy punch bleeding model

Group	Animals (*n*)	Defect sites (*n*)	Primary endpoint	Secondary endpoints
Control	3^a^	6	TTH	—Number of product applications needed to achieve hemostasis —Amount (%) of product needed to achieve hemostasis
Surgiflo		31		
Hemoblast		30		

*TTH:* time to hemostasis

^a^Twenty-two to 24 biopsy punch bleeding sites per pig and 3 additional sites in a fourth animal were created for a total of 30 Hemoblast-treated and 31 Surgiflo-treated sites. The first and last defect sites were used as untreated, control sites in each animal. All other sites were randomly assigned to Surgiflo or Hemoblast prior to treatment. Defect sites were created and treated sequentially ensuring that hemostasis was achieved before proceeding to create the next biopsy punch site

### Experimental bleeding sites

#### Treated sites

**Fig. 2:**
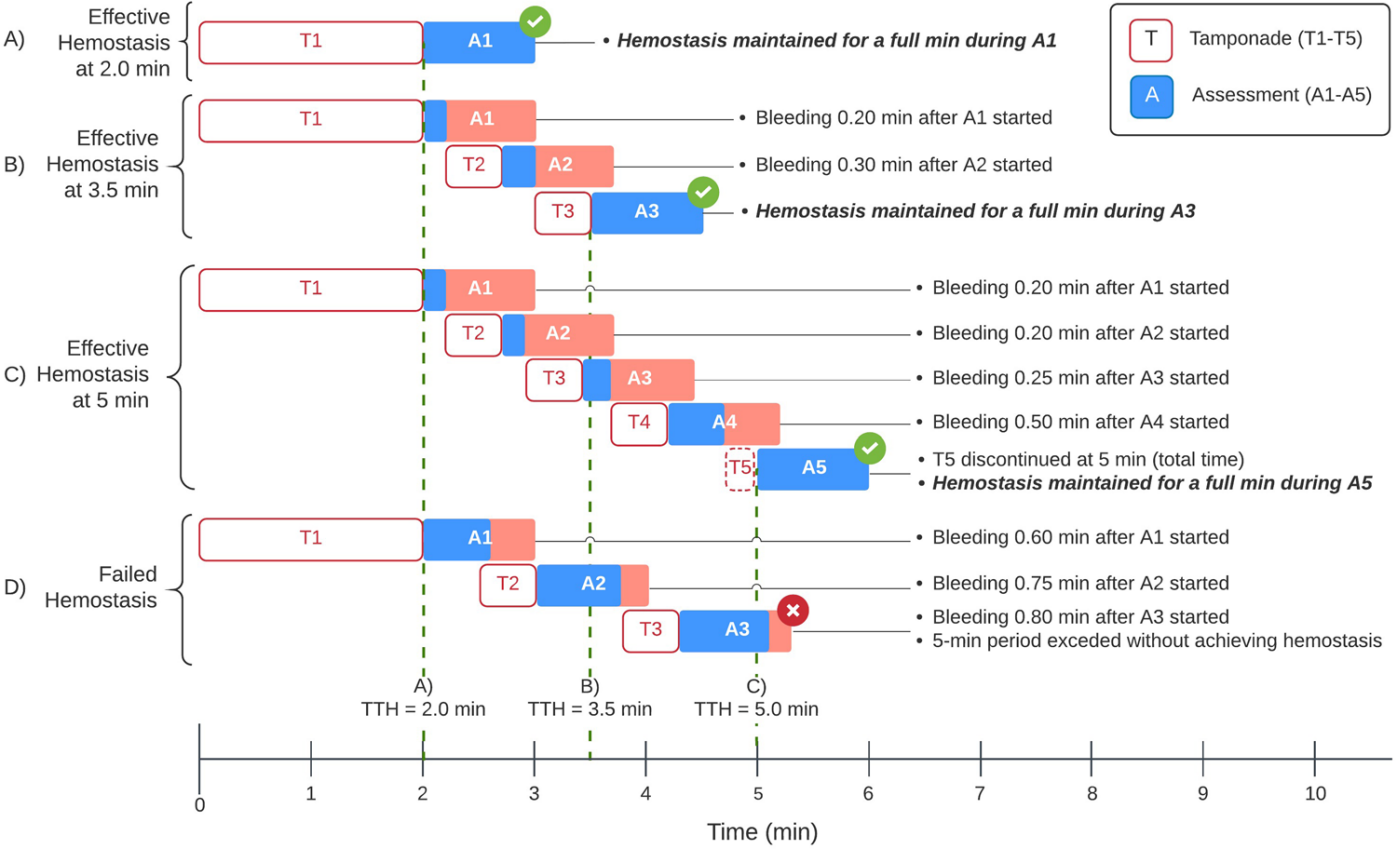
Sequence of tamponade (T) and visual assessment (A) periods after a randomly assigned product (Surgiflo or Hemoblast) is applied on an experimentally created biopsy defect. Three representative examples of effective hemostasis (**A**–**C**) and one of failed hemostasis (**D**) are depicted. While T1 is consistently applied for 2 min, each subsequent T period (T2–T5) lasts 0.5 min. Each A period may last up to 1 min. Only untreated, control defect sites received 6 T (not shown). Effective hemostasis was achieved and recorded as time to hemostasis(TTH) at 2, 3.5 and 5 min in the 3 examples shown (A, B and C, respectively). If the allotted 5-min period expires during a T application, as in the case shown in C, pressure is discontinued, and the defect site evaluated for a full min. If no bleeding is observed, the site is recorded as hemostatic with a TTH of 5 min. Panel **D** shows a case where 3 tamponades (T1-T3) are applied. Since the total 5-min period expired and the site did not achieve hemostasis for a full min during A3 (it started bleeding 0.8 min after A3 started), the site was declared failed hemostasis

Test material was applied to a freshly created lesion to cover the entire bleeding surface followed by the application of a saline-soaked gauze and manual pressure (tamponade) for 2 min (Fig. 1). Tamponade was then discontinued, the saline-soaked gauze was carefully removed, and the site was observed for any free-flow bleeding. If active bleeding stopped following the initial 2-min period of tamponade and hemostasis was maintained for a 1-min assessment period, the site was classified as effectively hemostatic and testing continued for a newly created bleeding site. However, if active bleeding was observed at any time during the first 60-s evaluation period, additional test material and tamponade were immediately reapplied for an additional 30-s period (Fig. 2). This subsequent 30-s treatment and up to 60-s observation period were repeated until hemostasis was achieved or the 5-min endpoint was reached without achieving hemostasis. Time to hemostasis (TTH) was defined as the time at which the last period of tamponade was discontinued for those sites classified as effectively hemostatic. If hemostasis was not achieved within 5 min, the test was declared a failure and TTH recorded as longer than 5 min. Figure 2 depicts the procedure following the initial product and tamponade application, examples of how TTH was determined for a defect site requiring multiple tamponade applications, as well as a case of failed hemostasis. If the 5-min period was completed while applying tamponade, pressure was stopped, and the site evaluated for up to 1 min. If no bleeding was observed during that 1 min observation period, the site was recorded as hemostatic with a TTH of 5 min (Fig. 2).

#### Control sites

For each of the 3 animals with 22–24 defect sites, 2 untreated, negative control sites were evaluated before the first treatment and after the last treatment protocol were completed to test whether hemostasis could be achieved by conventional methods. In these sites, only saline-soaked gauzes followed by digital pressure were applied. No splenic control defects were evaluated in the fourth animal before and after the first and last treatment, respectively; however, subsequent negative control liver abrasion lesions created for further testing in this pig did not achieve hemostasis after 5 min. Therefore, this approach confirmed that tamponade alone was not enough to control bleeding in physiologically and hemodynamically stable animals, and thus validated the in vivo model by demonstrating consistency across all treated sites between the first and last untreated lesions. Bleeding from sites that were not hemostatic after the 5-min observation period was controlled by applying another adjunctive topical hemostat (SURGICEL^®^ SNoW™ Absorbable Hemostat, Ethicon, Inc., Somerville, New Jersey, USA).

### Study design and statistics

Four animals were used and a total of 67 spleen biopsies were created for testing, including 30 bleeding sites for Hemoblast and 31 for Surgiflo, as well as 6 untreated, control sites from 3 pigs. Prior to treatment, spleen bleeding sites for each animal were randomly assigned to one of the two test materials (Surgiflo or Hemoblast) following a block randomization method to ensure a balanced sample size. Randomization was performed using a randomization function (Rand()) in Microsoft Excel. The surgeon was blind to treatment until after the lesion was created and scored for bleeding intensity. The primary endpoints of the study were hemostatic efficacy within 5 min and TTH. Predefined secondary endpoints included the number of test material applications needed, and the percent of test material per device applied on each site. In addition, the total amount of thrombin applied per defect site for each treatment was estimated from the percent of test material per device applied and the known amount of thrombin present in each full device.

Based on historical data from a small pilot study with Surgiflo and Hemoblast, 30 samples per group were performed in order to provide over 80% power to evaluate the non-inferiority of Surgiflo vs. Hemoblast assuming success rates of 95% and 85%, respectively. Descriptive statistics for the variables reported include sample median and 95% confidence interval (CI) and mean ± standard deviation (mean ± SD). Hypothesis testing was performed for the primary endpoints (hemostatic efficacy and TTH). We tested the alternative hypotheses (H_a_) that the rate of hemostasis at 5 min for Surgiflo treatment was non-inferior to that of Hemoblast with a delta (non-inferiority margin) of −0.15 (Test 1: H_o_: Surgiflo − Hemoblast ≤ −0.15; H_a_: Surgiflo − Hemoblast > −0.15) and superior to that of Hemoblast (Test 2: H_o_: Surgiflo − Hemoblast ≤ 0; H_a_: Surgiflo − Hemoblast > 0). A bootstrap method was used to calculate *p*-values for hypothesis Test 1 (non-inferiority) and hypothesis Test 2 (superiority). Sample size was calculated by simulating the bootstrap method 10,000 times using the assumed rates. A third test (Test 3), the log-rank distribution of the differences between TTH for the 2 treatments, was also estimated. Differences were considered significant when the *p*-value was less than 0.05. A gatekeeping method was used to maintain the overall, family-wise error rate at 5%. Test 2 was performed only if the null hypothesis (H_o_) of Test 1 was rejected. Test 3 was performed only if the null hypothesis of Test 2 was rejected. Median event time—TTH within 5 min 95% CI, and hemostasis distribution curves (Kaplan–Meier curves) for the two treatments within the first 5 min after creating a bleeding lesion are reported. If hemostasis was not achieved at 5 min in each lesion site—ineffective hemostasis—the site was censored at 5 min. Secondary endpoints and total thrombin applied per site were reported as mean ± SD values. A *post hoc* analysis of the percent of test material per device applied on each site and the total amount of thrombin applied per defect site for each treatment was performed using two-tailed *t*-tests with a 0.05 significance level assuming unequal variances. Descriptive statistics and statistical tests were performed using R Studio Version 1.2.1335 [32].

## Results

### Hemostatic efficacy and TTH

**Fig. 3:**
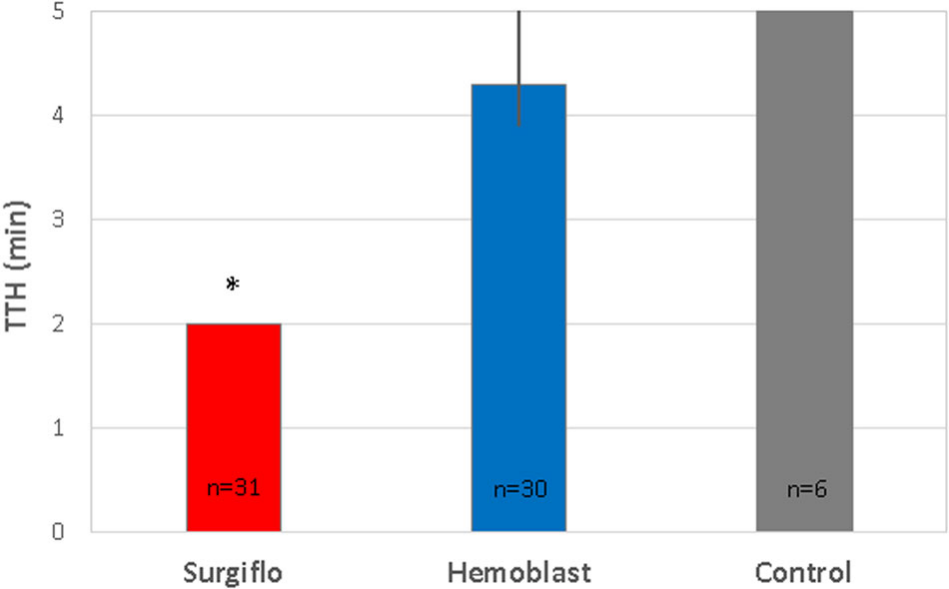
Time to hemostasis (TTH) in porcine spleen model of bleeding. TTH is expressed as median and 95% confidence interval (CI, open bar). Surgiflo required less than half the time (2.0 min, 95% CI [2.0, 2.0]) to achievehe mostasis compared to Hemoblast (4.3 min, 95% CI [3.9, 5.0]). Differences were statistically significant for noninferiority and superiority of Surgiflo vs. Hemoblast (*p < 0.01). All untreated, control sites failed to achieve hemostasis

**Fig. 4:**
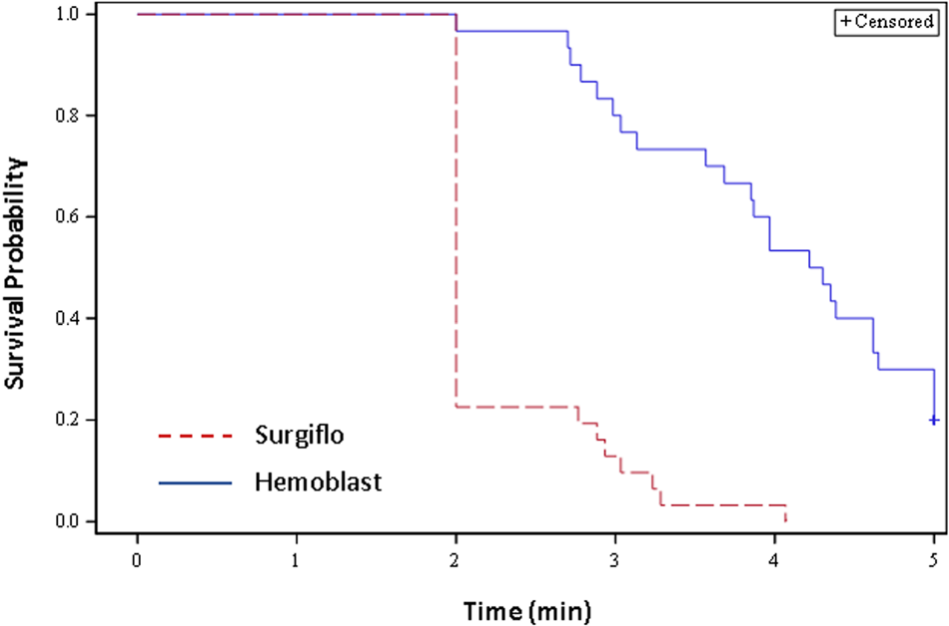
Product-Limit Survival Estimates (Kaplan–Meier [KM] curves). A log-rank test was used to compare the survival distributions of hemostasis between the Surgiflo and Hemoblast groups. A drop in the KM curve (low survival probability) indicates effective hemostasis, whereas high survival probability values indicate continued bleeding. A censored event is a site that continued bleeding after the 5-min observation period (ineffective hemostasis). The Hemoblast group showed a higher survival probability compared to the Surgiflo group. No censored events were recorded for Surgiflo, whereas some Hemoblast sites were censored

Seventy spleen defects were scored as moderate (98.6%, 70/71) and one as mild bleeding. No defects were scored as severe. Evaluation of control, untreated spleen biopsy sites confirmed that tamponade alone applied repeatedly over the 5-min treatment/observation period was insufficient to control blood loss. Hemostasis was achieved within 5 min for all sites treated with Surgiflo (31/31, 100%), and for 24 out of 30 of the Hemoblast-treated sites (80%) (Table 2). Median TTH was 2.0 min (95% CI: 2.0, 2.0) for Surgiflo-treated sites and 4.26 min (95% CI: 3.85, 5.00) for Hemoblast-treated sites (Fig. 3). The non-inferiority test (Test 1) was significant with a *p*-value of <0.01. Following the gatekeeping strategy, the superiority test (Test 2) was performed resulting in a *p*-value of <0.01. Thus, the hemostatic efficacy of Surgiflo was both non-inferior and superior to the hemostatic efficacy of Hemoblast (Table 2, Fig. 3). The log-rank test (Test 3) also resulted in a *p*-value of <0.01. The event (Kaplan–Meier) curves in Fig. 4 represent effective hemostasis (non-survival), and continued bleeding (survival). Events were censored if continued bleeding was observed after the allotted 5-min period. The Kaplan–Meier curves indicate that Surgiflo was a more effective hemostat than Hemoblast in this model.

**Table 2:** Number of defect sites that achieved effective hemostasis

	Defect sites (*n*)	Effective hemostasis sites^a^ (%)
Untreated	6	0 (0)
Surgiflo	31	31 (100)*
Hemoblast	30	24 (80)

*p-value < 0.01 vs. Hemoblast for non-inferiority and superiority

^a^Percentage of sites that achieved hemostasis within the 5-min treatment period. Surgiflo was more effective than Hemoblast

**Fig. 5:**
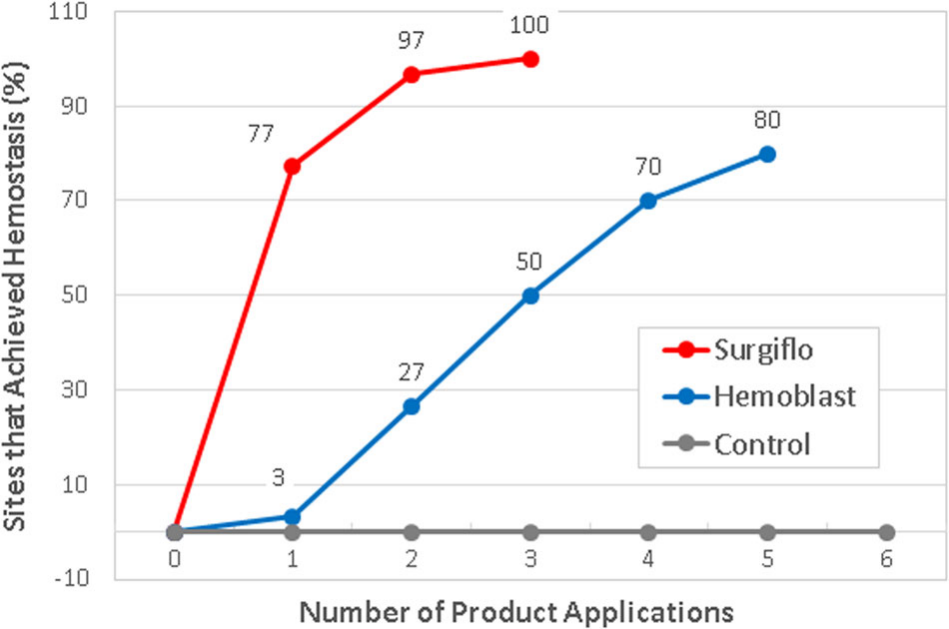
Cumulative percentage of sites that achieved hemostasis after 1–5 product applications. Twenty-four Surgiflo-treated sites (24/30, 77.4%) achieved hemostasis following 1 application, whereas only 1 (1/30, 3.3%) Hemoblast-treated sites achieved the hemostasis following a single product application. After 3 consecutive applications, 100% of Surgiflo-treated sites were hemostatic compared to 50% of Hemoblast-treated sites. 30% and 20% of splenic sites treated with Hemoblast did not achieve hemostasis after 4 and 5 applications, respectively. All Control sites failed as they were not hemostatic after the 5-min observation period and up to 6 tamponade applications. Bleeding of Control sites was stopped by applying another adjunctive topical hemostat (SURGICEL® SNoW™ Absorbable Hemostat)

The cumulative percentage of sites that achieved hemostasis following 1–5 product applications is shown in Fig. 5. Following the first product application, 77.4% of Surgiflo-treated splenic sites (24/30) achieved hemostasis, whereas only 3.3% of Hemoblast-treated sites (1/30) had attained a similar hemostatic outcome. After 3–4 product applications, 96.8% and 100% of Surgiflo-treated defect sites were hemostatic, whereas only 50.0% and 70.0% of Hemoblast-treated sites reached effective hemostasis, respectively. Twenty percent of splenic sites treated with Hemoblast did not achieve hemostasis within the allotted 5-min period even after up to five applications (Fig. 5).

### Product application

**Fig. 6:**
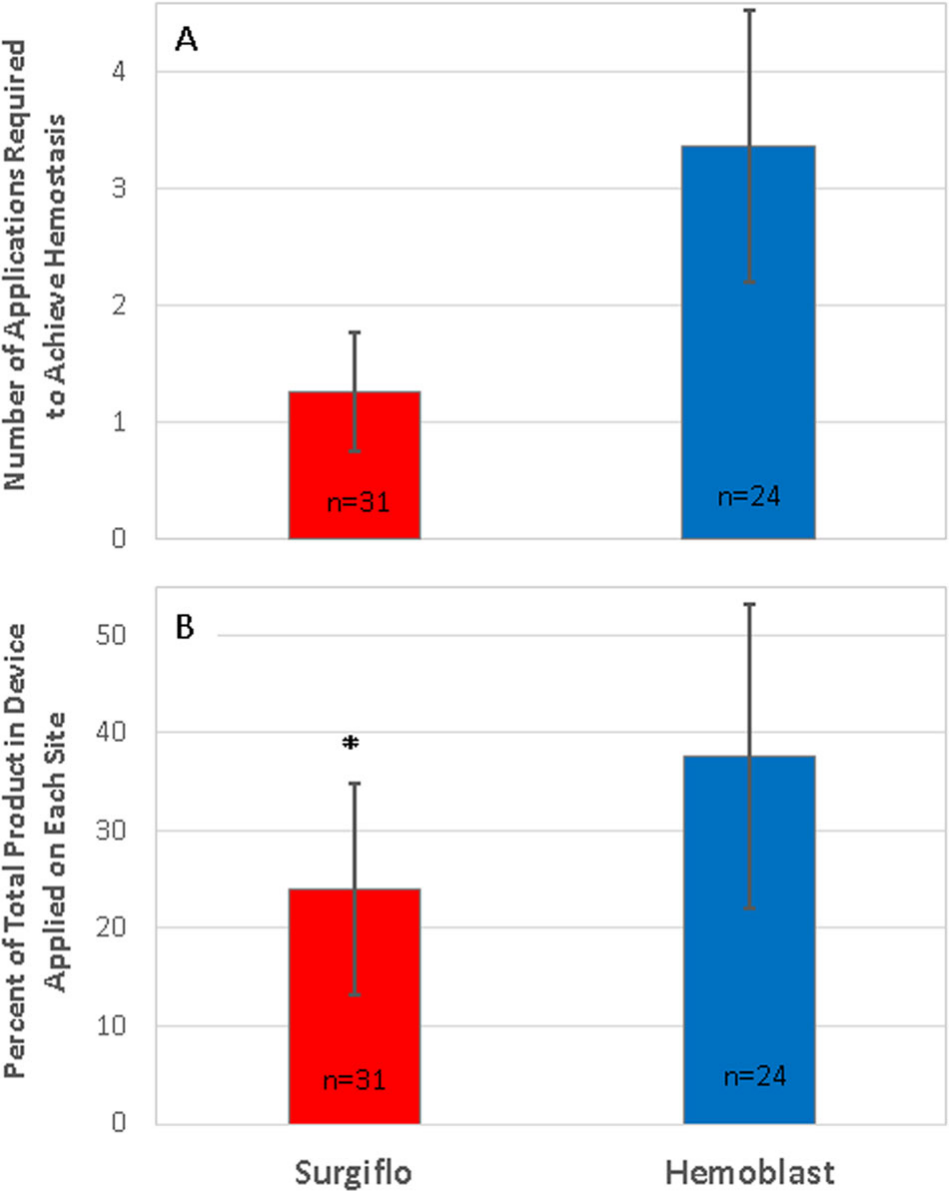
Product application per site for sites that achieved hemostasis. **A** Average number of product applications required to achieve hemostasis. Hemoblast required more than twice the number of applications than Surgiflo to achieve hemostasis (3.37 ± 1.16 vs. 1.26 ± 0.51). **B** Percent of total product per device applied on each lesion site. Surgiflo-treated sites required less product per device to achieve hemostasis compared to sites treated with Hemoblast (24.1 ± 10.8 vs. 37.6 ± 15.5% of total product contained in device). Data expressed as mean ± SD. *p < 0.001

The number of product applications and the total amount of product applied were recorded for all splenic lesion sites. The average number of product applications required during the up to 5-min allotted period was 1.26 ± 0.51 (mean ± SD) applications for Surgiflo and 3.37 ± 1.16 for Hemoblast (Fig. 6A). The applied product lifted off in 4 out of the 30 Hemoblast-treated sites and was subsequently found to be ineffective at achieving hemostasis in 2 of those sites. No such events of product disruption were recorded for Surgiflo-treated sites. The amount of product applied to the lesion sites is reported in Fig. 6B as a percent of the total mass provided in a single device. Surgiflo-treated sites received an average of 24.1 ± 10.8% material of a full device, whereas an average of 37.6 ± 15.5% material was applied to Hemoblast-treated sites (*post hoc* *t*-test *p* < 0.001). Based on the percent of product per device applied on each site and the known thrombin concentration in each device, the average (mean ± SD) amount of thrombin applied was estimated for Surgiflo-treated sites (482 ± 217 IU) and Hemoblast-treated sites (564 ± 232 IU). Differences between the means of the two groups were not statistically significant (*post hoc* *t*-test *p* = 0.159).

## Discussion

Tissue disruption during surgical procedures and trauma leads to bleeding that interferes with visualization of the surgical area and blood loss that may require replacement with blood or blood products. Trauma-induced hemorrhagic shock is a major cause of morbidity and mortality [33], and bleeding-related complications resulting from surgeries and endovascular interventions has been linked to longer length of hospital stays and higher cost of care for a variety of surgical procedures including neurological, vascular, solid organ, thoracic, cardiac, digestive and reproductive organs, and knee/hip replacements [8, 34–39]. Pre-hospitalization and inpatient management of bleeding during surgical procedures is crucial to prevent nonfatal and potentially life-threatening complications [40, 41]. Methods for controlling surgical bleeding have evolved from conventional mechanical means (e.g., tamponade, ligation, suturing, or electrocautery) to the use of adjunctive agents that accelerate hemostasis when conventional methods are considered ineffective or impractical to use. Hence, the development of more efficacious topical hemostatic agents to improve surgical outcomes has become an important objective to facilitate hemostasis and reduce the risk of bleeding-related complications.

Surgiflo is a flowable formulation especially useful for achieving hemostats in difficult to access anatomical locations. The flowable matrix can be delivered rapidly and moderate amounts of dispensed material can remain in place potentially reducing bleeding-associated complications. Reabsorption of gelatin-based flowable matrices usually occurs within 2–6 weeks depending on the extent of tissue vascularization, remaining amount of hemostat and tissue hydration rate [31, 42]. The original formulation of Surgiflo without added human thrombin [26] has been tested in a wide array of surgeries including neurosurgical procedures [15], salivary gland excisions [43], and cardiac surgeries [44]. Attaining efficacious hemostasis in cranial and spinal surgery is particularly relevant because the use of electrocautery carries the risk of destroying vital structures, e.g., nervous tissue resulting in neurological sequelae [45]. In a recent retrospective study that compared Floseal^®^ (Baxter Healthcare, Deerfield, IL, USA) and Surgiflo, both gelatin hemostatic matrices, in a consecutive series of 318 patients scheduled for elective and emergency cranial surgery suggested that both agents had similar efficacy, highlighting the value of gelatin-based hemostatic adjuncts in neurological procedures [45]. Another study reported the safe use of Surgiflo delivered into the epidural space to assist in hemostasis during spinal surgery [15]. A case series of submandibular gland excisions also reported the safe use of Surgiflo in place of a surgical drain [43]. Surgiflo has been successfully used after endoscopic sinus surgery [46] and to manage recurrent and post-traumatic epistaxis [47, 48]. As a more recently introduced product, there are fewer clinical reports describing the use of Hemoblast than there are for Surgiflo. However, a small (105 subjects), multicenter, randomized clinical trial of nonemergent cardiothoracic procedures showed that Hemoblast was superior to Floseal Hemostatic Matrix for total TTH, a measure of TTH that includes the time required to prepare the device, at 3 and 5 min [49]. Hemoblast is a ready-to-use powder that does not require mixing of components, thus eliminating preparation time before application as required by Floseal, Surgiflo, and some other commercially available products [19, 21, 50].

The current study represents the first evaluation of the hemostatic efficacy of Surgiflo and Hemoblast in a large animal model that consistently produced splenic lesions exhibiting moderate bleeding (98.6% of punch biopsy defects). Surgiflo was superior to Hemoblast in every primary endpoint examined in the porcine spleen punch biopsy model used in this study. Surgiflo demonstrated superior hemostatic efficacy and faster TTH compared to Hemoblast after 1–5 product applications. Twenty percent of Hemoblast-treated sites failed to achieve hemostasis after five applications, and Hemoblast required twice as many applications as Surgiflo to achieve hemostasis within the allotted 5-min period. Large efficacy gaps were observed between Surgiflo and Hemoblast after each application. The efficacy gap observed between the two hemostats decreased progressively as the number of applications increased. An initial efficacy gap of 74% (77.4–3.3%) was reduced to 70%, 50%, and 30% after 2, 3, and 4 product applications, respectively (Fig. 5). Although no additional preclinical studies comparing Surgiflo and Hemoblast have been published, Floseal, another commercially available gelatin flowable matrix with thrombin, has been compared to Hemoblast in a porcine liver bleeding model [51]. In that study, both products demonstrated hemostatic efficacy for controlling mild to moderate bleeding. Mild or moderate bleeding rates were lower for Floseal compared to Hemoblast at 3 and 7 min. No statistically significant difference in mild or moderate bleeding rates was found at 11 min between the two hemostats.

Data from a porcine spleen model conducted by our research group indicated that Floseal and Surgiflo, both gelatin-based matrices, demonstrated similar hemostatic efficacy when compared to control for mild and moderate bleeding [31]. However, a morphological study comparing the hemostatic efficacy of Floseal and Surgiflo in a porcine liver trauma model [52] indicated that Floseal provided superior control of bleeding than Surgiflo. However, it is noteworthy that the authors were using a subjective scoring method. The morphological report in question [52] suffers from serious experimental design limitations. Briefly, the effects of Floseal and Surgiflo were compared in two pigs referred to as Piglet 1 and Piglet 2. Piglet 1 was presumably kept under “Physiological Conditions”, whereas Piglet 2 was subjected to “Hypotension, Hypothermia, and Hypovolemia”. It is unclear how the experimental hypotensive, hypothermic, and hypovolemic states were induced in Piglet 2. Since a single pig was used per group/condition(s), no statistical analysis could be conducted. Although the authors claim that Piglet 2 was hemodiluted—9 L of fluids were administered intravenously over a 3-h period—no hematocrit values were provided for either animal to assess the degree of hemodilution. Moreover, Piglet 1 also received 7 L of fluid, presumably over the same 3-h period. Additionally, the hypovolemic animal (Piglet 2) required “inotropic support”. The inotropic agents—epinephrine and norepinephrine—administered at non-specified doses may have affected not only myocardial contractility but also vascular reactivity. In several species, the liver vasculature responds with vasoconstriction to norepinephrine via alpha-adrenoceptors, reducing hepatic blood flow [53]. Furthermore, hepatic blood flow exhibits regional heterogeneity, which is affected by norepinephrine [54]. Therefore, administration of catecholamines may have affected blood flow—and the degree of bleeding from the experimentally induced wounds—differently in the three porcine liver lobes. Four products (Surgiflo and Floseal with and without thrombin) were randomly applied to four hepatic lesions per liver lobe and one lesion was used as control. Taken together, the absence of relevant quantitative hemostasis-related measurements, the subjective nature of the hemostasis scoring method used, the unnecessary complexity of the experimental design (two groups and four independent treatments per liver lobe), the sample size constraint (*n* = 1 animal per group) and lack of statistical analysis, the apparently profound changes in hemodynamics and cardiovascular physiology experienced by the animals, and the nonquantitative nature of the morphological data presented prevent drawing any conclusion from this report regarding efficacy of the products tested.

Surgiflo, when used with thrombin, and Hemoblast are both ‘active’ hemostats because they include thrombin in their formulation. As a component of the clotting cascade involved in proteolytic steps leading to the formation of crosslinked fibrin, thrombin is added to topical hemostats to have an ancillary effect on clot formation. In the current study, exploratory post-hoc comparisons of the secondary endpoints revealed statistically significant differences in the total product mass per device required to achieve hemostasis. Surgiflo-treated sites achieved hemostasis with fewer product applications, and significantly less product per device compared to Hemoblast-treated lesions. However, differences between the average amount of thrombin applied in Surgiflo- and Hemoblast-treated defects were not statistically significant, suggesting that the superior hemostatic efficacy of Surgiflo in the spleen punch biopsy model used was primarily due to the properties of Surgiflo matrix (porcine gelatin). Although the amount of thrombin applied per site was a secondary endpoint not defined in the protocol, it was estimated from the percent of product per device applied on each defect site, a predefined secondary endpoint. Both variables correlated perfectly (*r*^2^ = 1). Notwithstanding, lack of differences between Surgiflo and Hemoblast regarding the amount of thrombin applied per site should be interpreted cautiously.

The spleen was selected in this model as it is a densely vascularized lymphoid organ [55–57]. The moderate bleeding that results from experimental lesions in the spleen punch biopsy model we used differs from other models (e.g., hepatic abrasion) in that spleen lacerations and punch biopsy defects result in continuous flowing of blood due to an extensive damage of a dense network of capillaries and small vessels subjacent to the lesion. Flowable hemostatic agents such as Surgiflo create a blockade to blood flow and provide a surface on which blood clots [58]. Although Surgiflo and Hemoblast share a mechanism of action because of the presence of collagen and gelatin-based matrices and thrombin in both formulations, Surgiflo is a flowable product exhibiting higher density and consistency compared to Hemoblast, which is a powder agent. While flowable hemostats can be applied deep into surgical wounds providing a strong barrier to lesions with moderate flowing bleeding, powder hemostats do not possess enough physical cohesiveness and density to penetrate and stop flowing bleeding. Thus, topical hemostats in a powdered form may be more effective at stopping diffuse mild bleeding, whereas flowable hemostats are more effective at achieving fast and sustained hemostasis in lesions with moderate bleeding [58]. Hemoblast’s ready-to-use formulation is a valuable feature in a surgical setting, as it requires no special preparation. Nonetheless, Surgiflo gelatin matrix and thrombin components can be rapidly and easily mixed within a minute. Furthermore, once prepared, Surgiflo can be stored at room temperature and used for up to 8 h without losing consistency or fluidity.

A large observational retrospective clinical study from 121 hospitals and a total of 24,882 records found that the cost savings associated to the use of Surgiflo were considerably higher compared to Floseal [59], and a retrospective analysis of cardiac surgical cases (2006–2012, from Premier US Perspective Hospital Database) in which Floseal or Surgiflo were used, reported no statistically significant differences in mortality or length of stay between the two flowable hemostats [44]. Results from that study suggested that cases where Surgiflo was used were associated with significantly higher risk of adverse outcomes [44]. It should be noted that sample sizes of records examined retrospectively were more than 10 times larger for all Floseal groups compared to the Surgiflo groups. Since Floseal has been in the market longer than Surgiflo, cases treated with Floseal exceeded those treated with Surgiflo. As stated by the authors, no adjustments were applied to the datasets to correct for the large differences in sample sizes between the groups and subgroups. It is well accepted that the use of unbalanced sample sizes may lead to unequal variances, which in turn affect statistical power and Type I error rates [60]. Therefore, it remains to be ascertained whether equivalence testing and further retrospective analyses of the datasets using balanced sample sizes would render similar results. Additionally, the original Surgiflo formulation was used in all cases examined. The Surgiflo formulation tested in the present study contains thrombin and exhibits similar hemostatic efficacy compared to the original formulation, and it has been shown to be an effective hemostat at controlling bleeding during various surgical procedures including tonsillectomy and adenoidectomy [61] and posterior lumbar surgery [62].

## Study limitations

Our study was randomized for the articles tested and the surgeon was blinded to treatment identity until the bleeding classification was revealed and recorded. The physical appearance of the devices prevented the surgical team from masking device identity and thus conducting a blinded study. Hemoblast IFU instructs users to maintain the product over the bleeding site using wound pressure and a saline-soaked gauze for at least 2 min when using the CE Marking product (EEA) used in the present study [24], or ~3 min when using the FDA-approved product (US) [27]. Surgiflo IFU recommends to inspect the wound site after 1–2 min [23]. TTH was evaluated at 2 min followed by additional product applications if hemostasis was not achieved within the 5-min period. The 2-min procedural assessment time followed by product reapplication—and 30-s reassessment periods—if hemostasis was not achieved, reflects real-world scenarios faced by surgical teams treating mild to moderate hemorrhage.

Numerous potential confounding factors in the design of animal study protocols have been reported [63]. Our study design controlled for many variables including pig strain, age, sex and weight, husbandry techniques, feeding (timing, amount), oral fluid intake, animal environment, randomization method, and blinding to treatment. Our experimental protocol was intended to provide a scientifically sound and reproducible model to allow for reliable comparisons of adjunctive hemostats. However, differences in coagulation and hemostasis may exist between male and female pigs, and lack of inclusion of male animals may have affected the study outcome.

## Conclusion

The present study has demonstrated that Surgiflo, a flowable gelatin matrix product containing human thrombin, is a more effective adjunctive topical hemostat for moderate organ bleeding than Hemoblast, a powder matrix containing collagen, chondroitin sulfate and human thrombin. Surgiflo demonstrated superior hemostatic efficacy for moderate bleeding in a well-established and reliable porcine spleen model for hemostasis assessment. Surgiflo required a single 2-min application to achieve hemostasis in nearly 3 quarters of the sites treated. Surgiflo is a highly effective flowable adjunctive hemostat and a better-suited tool compared to Hemoblast, a powder agent, for surgical teams aiming at controlling continuous, flowing bleeding from lesion sites where conventional methods are ineffective or impractical to use. Differences between both hemostats were independent of the amount of thrombin applied suggesting that the superior hemostatic efficacy of Surgiflo in the porcine spleen punch biopsy model was due to Surgiflo matrix properties as a malleable barrier that was able to adjust to defect topography and provide a surface for effective blood clot formation, while thrombin provided an ancillary effect.
